# 3D Printed Carbon Nanotube/Phenolic Composites for Thermal Dissipation and Electromagnetic Interference Shielding

**DOI:** 10.1021/acsami.4c17115

**Published:** 2024-12-04

**Authors:** Thang Q. Tran, Sayyam Deshpande, Smita Shivraj Dasari, Kailash Arole, Denis Johnson, Yufan Zhang, Ethan M. Harkin, Abdoulaye Djire, Hang Li Seet, Sharon Mui Ling Nai, Micah J. Green

**Affiliations:** † Artie McFerrin Department of Chemical Engineering, Texas A&M University, College Station, Texas 77843, United States; ‡ Singapore Institute of Manufacturing Technology (SIMTech), Agency for Science, Technology and Research (A*STAR), 5 Cleantech Loop, #01-01 Cleantech Two Block B, Singapore 636732, Republic of Singapore; § Department of Materials Science and Engineering, Texas A&M University, College Station, Texas 77843, United States

**Keywords:** phenolic resin, carbon nanotube, direct ink writing, electromagnetic interference shielding, heat dissipation

## Abstract

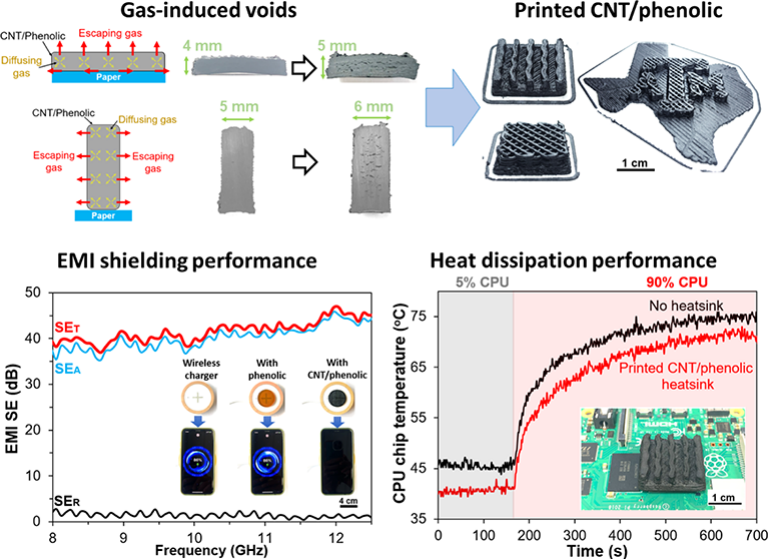

Here we demonstrate direct ink write (DIW) additive manufacturing of carbon nanotube (CNT)/phenolic composites with heat dissipation and excellent electromagnetic interference (EMI) shielding capabilities without curing-induced deformation. Such polymer composites are valuable for protecting electronic devices from overheating and electromagnetic interference. CNTs were used as a multifunctional nanofiller to improve electrical and thermal conductivity, printability, stability during curing, and EMI shielding performance of CNT/phenolic composites. Different CNT loadings, curing conditions, substrate types, and sample sizes were explored to minimize the negative effects of the byproducts released from the cross-linking reactions of phenolic on the printed shape integrity. At a CNT loading of 10 wt %, a slow curing cycle enables us to cure printed thin-walled CNT/phenolic composites with highly dense structures; such structures are impossible without a filler. Moreover, the electrical conductivity of the printed 10 wt % CNT/phenolic composites increased by orders of magnitude due to CNT percolation, while an improvement of 92% in thermal conductivity was achieved over the neat phenolic. EMI shielding effectiveness of the printed CNT/phenolic composites reaches 41.6 dB at the same CNT loading, offering a shielding efficiency of 99.99%. The results indicate that high CNT loading, a slow curing cycle, flexible substrates, and one thin sample dimension are the key factors to produce high-performance 3D-printed CNT/phenolic composites to address the overheating and EMI issues of modern electronic devices.

## 1 Introduction

With the development of artificial intelligence and 5G communication technologies in recent years, modern electronics have become smaller, faster, and more highly integrated.^1−3^ Consequently, electronic devices require significant increase in high-power density, leading to overheating.^4,5^ Additionally, such devices have become more sensitive to external electromagnetic (EM) signals.^6−9^ In some cases, they can even become new sources of EM pollution to other surrounding electronic components as well as the nearby human and natural environment.^8,10^ Overheating and EMI issues can lower the performance, reliability, and shelf life of the electronic devices.^10,11^ Therefore, multifunctional thermal management (TM) materials with both high electromagnetic interference (EMI) shielding effectiveness and high thermal conductivity are desirable to meet the requirements of next-generation electronic devices,^9,12,13^

Although traditional metals can serve as good EMI shielding and TM materials, they possess several disadvantages, such as difficult processability, heavy weight, and high cost, which make them less suitable for modern electronic devices.^14,15^ Moreover, due to high electrical conductivity, the dominant EMI shielding mechanism for metals is reflection.^14^ Therefore, rather than eliminating incident EM waves, they mainly reflect these waves back into the environment.^14^

In contrast, polymer nanocomposites embedded with conductive fillers have drawn significant attention in the field of TM and EMI shielding materials due to their properties, such as low density, corrosion and chemical resistance, low cost, and good processability.^11,13,16,17^ By constructing different polymer nanocomposite structures with multiple interfaces, multiple reflection or scattering of EM waves at interfaces and interfacial polarization can be achieved, resulting in high EMI shielding efficiency with very low reflection.^18−20^ Additionally, the filler loading of the polymer composites can be tailored to achieve not only good heat dissipation capability but also high EMI shielding performance with low EM pollution,^9,12,13^

Additive manufacturing (AM), i.e., 3D printing, technologies are advanced techniques that can effectively process polymer composites with less material waste and easier customization compared to conventional manufacturing processes.^21−27^ Moreover, the sequential layer deposition approach of AM offers the ability to fabricate polymer composite parts with complex shapes and high surface area to effectively address the overheating and EMI shielding issues.^11,28,29^ In fact, many studies have successfully applied different 3D printing techniques to process polymer composites for TM,^4,11,29^ and/or EMI shielding applications.^8,9,14^ In these studies, thermally and electrically conductive fillers, such as carbon nanotubes,^6,11^ carbon fibers,^29,30^ and graphene nanoplatelets,^8,9^ are often used to modify the AM polymer feedstocks for desired printability and/or enhanced thermal dissipation and EMI shielding performance. Additionally, 3D printing can be combined with conventional production methods to fabricate unique polymer composite structures for enhanced EMI shielding property. For example, Hou et al. fabricated a ring-shaped electromagnetic synergistic structure made of polylactic acid, carbon nanotubes (CNTs), polydimethylsiloxane, and carbonyl iron by combining fused filament fabrication and solution casting.^31^ Due to the magnetic loss, conduction loss, and reflection loss resulted from the unique structure, their polymer nanocomposites exhibited an excellent EMI shielding property with very low EM pollution.^31^

Here, we focus on a useful but difficult polymeric resin: Phenolic resins are thermosets that possess several outstanding properties, such as low density, high strength-to-weight ratio, low cost, good heat resistance, excellent chemical and corrosion resistance, outstanding impact and fatigue properties, and stable performance.^32^ Consequently, they have been used as matrices for polymer composites in different applications, including electronics, military, aerospace, and automotive.^32^ However, studies on the use of phenolic composites for protecting electronic devices from overheating and EMI issues have yet to be reported. One main disadvantage of phenolic resins is the generation of water and gases during their curing reactions, leading to the formation of voids and cracks within the internal structure.^33−35^ To minimize these negative effects, the curing process of phenolic resins is usually conducted under increased pressure (by using composite autoclaves)^34^ or reduced pressure (by using vacuum ovens).^35^ However, this approach is not applicable to 3D-printed phenolic parts with complex geometries due to shape distortion caused by pressurized or vacuum conditions. In fact, there are very few studies on the 3D printing of phenolic composites,^33^ and the effects of different important factors, such as curing cycle, sample size, filler loading, and substrate types, on the internal structure of the 3D-printed phenolic composites have not been investigated yet.

In this paper, we fabricated carbon nanotube (CNT)/phenolic composites with good heat dissipation and high EMI shielding capabilities using direct ink writing (DIW) 3D printing. This is the first time that 3D-printed phenolic composites have been used for dual protection of electric devices from EMI and overheating issues. Here, CNTs are employed as a rheological modifier and a conductive nanofiller to enhance the printability as well as heat dissipation and EMI shielding capabilities of the phenolic composites. The curing process of the CNT/phenolic prints is conducted at atmospheric pressure to minimize structural distortion. Moreover, different curing conditions are applied to study effects of the curing speed on shape retention of the printed CNT/phenolic composites. Additionally, the effects of sample size, CNT loading, and substrate types on the mesostructures of the printed CNT/phenolic composites are investigated comprehensively. The results suggest that high CNT loading, slow curing process, flexible substrates, and at least one thin dimension are the key factors to produce 3D-printed CNT/phenolic composites with good print quality. This work offers an attractive strategy for fabricating phenolic composites with a high filler content to address the current challenges in EMI shielding and overheating issues facing cutting-edge electronic devices.

## 2 Materials and Methods

### 2.1 Materials and Formulation

The multiwalled carbon nanotubes (CNTs) functionalized with COOH were purchased from Cheap Tubes Inc. (USA). The resole-type phenolic resin (PLENCO 14670) was donated by Plastics Engineering Company (USA). The inks were prepared by mixing the phenolic resin with appropriate amount of CNTs using a planetary mixer (Thinky corporation, AR-100) at a speed of 2000 rpm in 2 min.

### 2.2 3D Printing of Phenolic Samples

The inks were loaded into a 15 cm^3^ syringe (Hyrel 3D, EMO-XT) and centrifuged at 5000 rpm for 10 min to degas. After the loaded syringe was mounted in a print head of a DIW printer (Hyrel 3D, Engine SR), the CNT/phenolic inks were deposited layer-by-layer to fabricate 3D printed samples at a layer thickness of 0.5 mm by using a 0.84 mm-diameter nozzle. Two types of substrates were used in the printing process: aluminum foil coated with polytetrafluoroethylene mold release (referred to as Al substrate) and water-repellant paper (Durable Documents, USA). Print paths were generated in G-code using Repetrel software (Hyrel 3D, USA).

### 2.3 Thermal Curing of Phenolic Samples

After the prints were fully built, they were transferred to a heating oven (Thermo Fisher, Isotemp Model 282A) for curing. Two main curing cycles were applied to cure the phenolic samples: a fast cycle and a slow cycle. In the fast cycle, samples were heated rapidly to 200 °C, held for 15 min, and then cooled to room temperature at the cooling rate of 0.6 °C/min. In the slow cycle, a stepwise cure was conducted from 60 to 150 °C. Each step varied from 10 to 30 °C with a hold of at least 3 h. More details on the slow curing cycle can be found in Table S2.

The resole-type phenolic resin used in this study can self-cross-link without the incorporation of a curing agent. According to the phenolic manufacturer, the cross-linking reactions of the phenolic resins consist of two stages:^36^ In the first stage, phenol reacts with methylene glycol to produce methylol phenol. For the second stage, methylol phenol can react with itself to form a longer chain methylol phenolic or dibenzyl ether. It also can react with phenol to form a methylene bridge. More details on the chemical reactions can be found in Table S1. Notably, water is formed as a byproduct in all cross-linking reactions.

### 2.4 Characterization

A stress-controlled rheometer (Anton Paar, MCR 301) equipped with a double-gap measuring system (DIN 54453) was used to characterize the rheological properties of the phenolic composite inks before the printing. The printability of the composite inks was evaluated by measuring their viscosity as a function of the shear rate (γ). An amplitude sweep test was performed at a shear strain between 0.1% and 100% to measure the ink storage modulus (*G*′) and loss modulus (*G*″) as a function of shear stress.

The cross sections of the printed phenolic composite samples were investigated by using an optical microscope (BX51, Olympus). The alternating current (AC) conductivities of the printed composite samples were measured at room temperature with a frequency range of 1–10 MHz by using a dielectric spectrometer (Novocontrol Technologies GmbH). Cryo-fracture surfaces of the printed phenolic composite samples were characterized with a field emission scanning electron microscope (FE-SEM, Model Quanta 600 FEG, FEI Company).

The thermal conductivity of the printed phenolic composite samples was measured using a thermal conductivity analyzer (Hot disk, TPS 2500S). Thermal dissipation capability of the phenolic composite samples was qualitatively characterized by employing an infrared camera (FLIR Systems, Inc., A655sc) to record the temperature of the sample surfaces after they were heated by a heating plate at 100 °C.

Thermogravimetric analysis (TGA, TA Instrument TGA5500) of the neat phenolic resin was performed in air from 25 to 850 °C at a heating rate of 10 °C/min. Differential scanning calorimetry (DSC) test of the resin was conducted using a TA Instrument DSC2000 in a nitrogen atmosphere, with the temperature ranging from 25 to 250 °C at the same heating rate.

### 2.5 Heatsink Performance

The performance of the printed CNT/phenolic heatsinks was evaluated by attaching the heatsinks to the top of a central processing unit (CPU) chip of a microcontroller board (Raspberry Pi 4, model B). Heat conduction across the interface between the heatsink and the CPU chip was improved by using 250-μm-thickness thermal tape (McMaster-Carr) as a thermal interface material. The change in chip temperature with time was then measured under natural convection by using a Python script.

### 2.6 EMI Shielding Performance

EMI shielding performance of the printed CNT/phenolic samples was evaluated by employing a vector network analyzer (P9373A, Keysight Technologies) with a WR-90 rectangular waveguide in *X*-band frequency range (8.2–12.4 GHz). Each testing sample was a cuboid specimen with a width of 20 mm and a length of 30 mm. The specimen thickness was varied between 1 and 5 mm to study its effects on the EMI shielding performance. After the EMI shielding tests were conducted, scattering parameters (S11 and S21) were measured and the total EMI shielding effectiveness (SE_T_), reflection loss (SE_R_), and absorption loss (SE_A_) were determined by using eqs 1–3.^7,14^
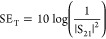
1
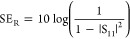
2
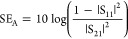
3

## 3 Results and Discussion

### 3.1 Rheology and Printing of CNT/Phenolic Inks

**Figure 1:**
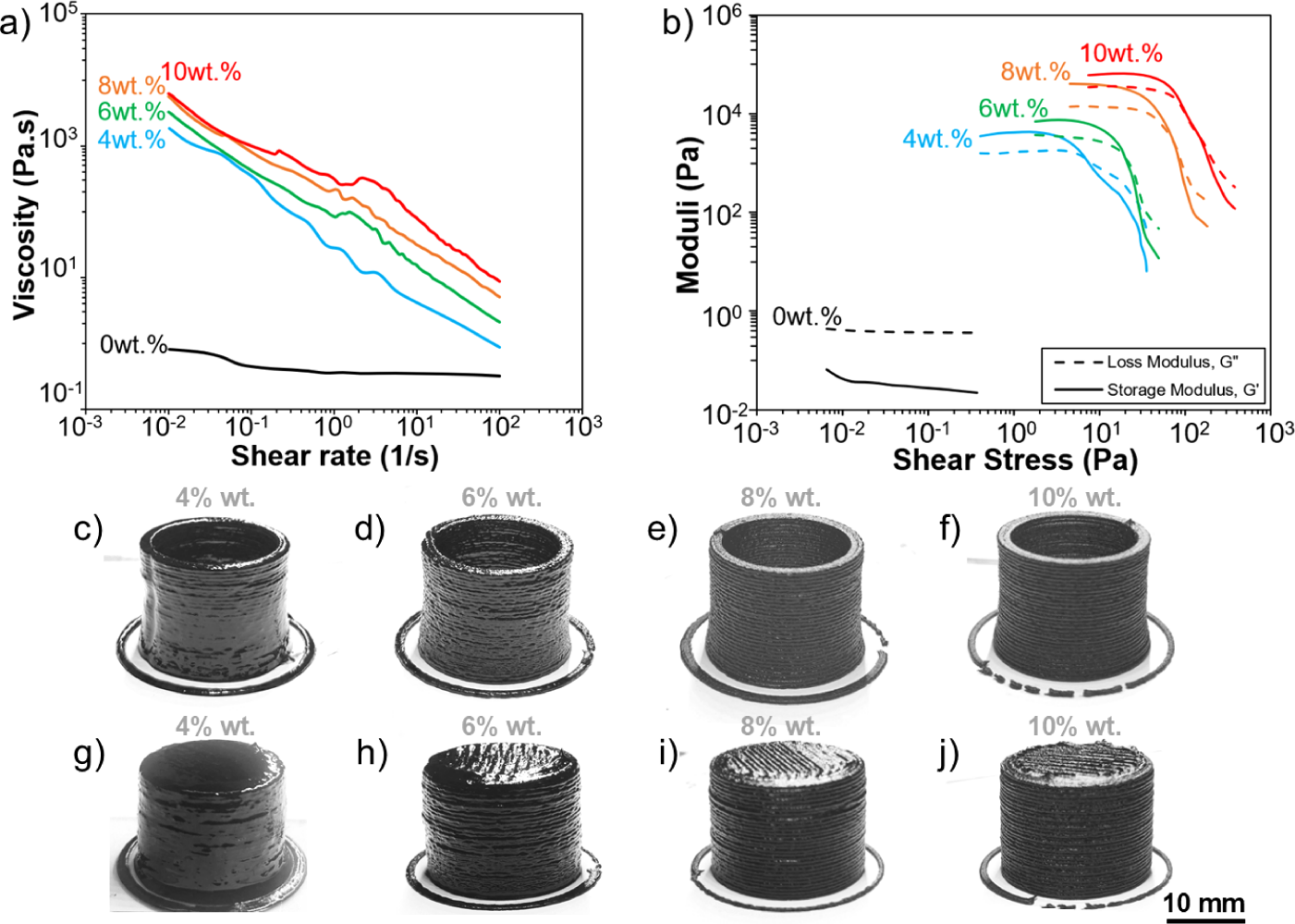
Plots of (a) apparent viscosity as a function of shear rate and (b) shear storage and loss moduli as a function of shear stress for CNT/phenolic inks at varying CNT loadings. (c–f) CNT/phenolic thin walls and (g–j) dense towers printed at different CNT loadings.

CNTs are employed to tailor the rheological properties and enhance the printability and cure stability of the phenolic composite ink formulations in this study. The rheological behavior of the phenolic-based inks at varying CNT loadings are shown in Figure 1a,b. The neat phenolic resin exhibited a viscosity of approximately 0.5 Pa·s, nearly independent of shear rate. Moreover, the shear loss modulus (*G*″) of the neat phenolic resin was higher than its storage modulus (*G*′). The findings indicate that the neat phenolic ink behaved like a liquid in the tested shear stress range.^11,22^ Therefore, although the neat resin can be easily extruded through the printing nozzle under modest applied pressures during the DIW printing process, a complete print cannot be achieved because the printed extrudate lacks the ability to support itself,^11,22,23^

However, the phenolic resin behaves as a viscoelastic fluid after integration of the CNT fillers. As shown in Figure 1a, the ink viscosity increased with increasing CNT loading across the tested shear rate range. Specifically, 4 wt % CNT/phenolic inks possessed a viscosity of 0.7 × 10^3^ Pa·s, whereas a viscosity of up to 0.6 × 10^4^ Pa·s could be achieved by inks with 10 wt % CNT loading at low shear rate (∼0.01 s^–1^). These results are orders of magnitude higher than those of the neat resin. Moreover, the viscosity of the CNT/phenolic composite inks reduced with an increasing shear rate, exhibiting strong shear-thinning behavior. At the shear rate typically associated with DIW printing (∼50 s^–1^), the apparent viscosities of the composite inks containing 4 wt % CNT and 10 wt % CNT were 1.5 Pa·s and 1.5 × 10^1^ Pa·s, respectively, which are only about an order of magnitude higher than that of the neat resin alone. Due to this shear-thinning behavior, the CNT/phenolic inks can be easily extruded through the print head nozzles without the need for excessive applied pressure during the DIW printing process.^11,22^

Similarly, the addition of the CNT fillers also resulted in a significant increase in the moduli and shear yield stress at the intersection of the storage and loss moduli curves of the composite inks. Specifically, when the loading of CNTs increased from 4 wt % to 10 wt %, the storage modulus of the composite inks below the yield stress increased to 7 × 10^4^ Pa, whereas their loss modulus reached 4 × 10^4^ Pa, corresponding to an increase by nearly 17.5 and 20 times, respectively. Similarly, the shear yield stress of the composite inks also increased from 7.5 to 97 Pa when CNT loading increased from 4 to 10 wt %. Notably, inks with higher yield stresses can maintain shape retention better once the inks are discharged from the printing nozzle,^11,22,23^

Figure 1c,d present CNT/phenolic thin walls and dense towers printed by DIW at different CNT loadings. All thin and dense structures can be printed with good shape retention and consistent dimension due to their sufficiently high yield stresses.^11,23^ At higher CNT loadings, the printed CNT/phenolic structures exhibited sharper surface features with more visible defined layers and filaments on the exterior surface.^11^

### 3.2 Effects of Curing Cycle on Shape Retention

Figure S1a shows the DSC curve of the neat phenolic resin at a heating rate of 10 °C/min. The first stage of the cross-linking reaction starts at ∼50 °C, peaks at ∼82 °C, and ends at ∼120 °C. The last stage starts right after that, peaks at ∼155 °C, and ends at ∼190–200 °C. The TGA curve of the phenolic resin (Figure S1b) was consistent with the DSC result with a total mass loss of 36% in the temperature range between 50 and 200 °C. The results suggest that all cross-linking reactions were completed at approximately 200 °C. Based on the findings, both fast and slow curing cycles were developed to cure the phenolic-based samples in this section.

**Figure 2:**
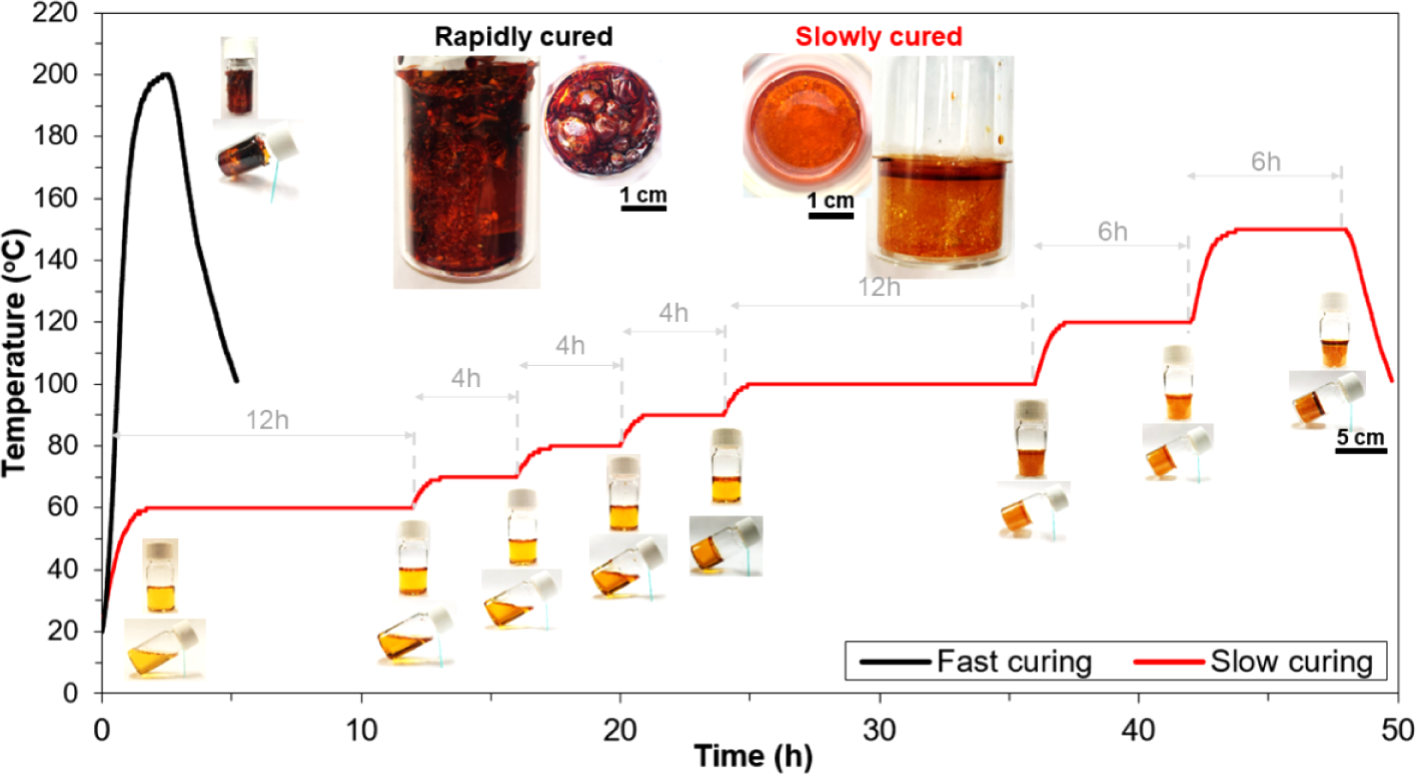
Fast and slow cycles for phenolic sample curing schedules (insets show neat phenolic resins in glass vials cured via fast and slow curing cycles).

Figure 2 shows temperature profiles of the fast and slow curing cycles that we selected to cure phenolic-based samples. In the fast-curing cycle, phenolic samples were rapidly heated to 200 °C and held for 15 min before being cooled down. As shown in the insets, the volume of the rapidly cured samples was significantly expanded, with many voids observed in their internal structures. Due to the high curing rate in the fast cycle, tremendous byproducts (mostly water vapor) were released in a short time, while phenolic resin changed rapidly from liquid to solid state. Consequently, a large amount of the byproducts could not escape from the samples, resulting in cured phenolic samples with severe void formation and shape distortion.^34^

For the slow cycle, phenolic samples followed a stepwise cure from 60 to 150 °C with an average heating rate of 0.2 °C/min. A long holding time of 12 h was conducted at 60 and 100 °C since these temperatures were the beginning of the two curing stages. At the first stage of the curing process (60 to 90 °C), the neat phenolic samples were still in liquid state and their viscosity increased gradually with increasing heating time and temperature, as shown in the insets of Figure 2. When heating the samples from 90 to 100 °C, the second stage of the curing process started, and the phenolic samples were slowly transformed from liquid to solid phase. Subsequently, a slightly faster curing process with fewer heating steps was applied to cure the samples due to the insignificant effects of the byproducts on the solid state of the samples. Since the highest reaction rate was achieved at around 150 °C, the phenolic samples were held at this temperature for 6 h to complete the slow curing cycle. Compared to the rapidly cured samples, the slowly cured samples had fewer voids and lower density changes. Due to the slow curing rate in the slow cycle, the liquid–solid state change occurred slowly such that most byproducts escape from the samples, resulting in reduced void formation and good shape integrity.^33−35^

**Figure 3:**
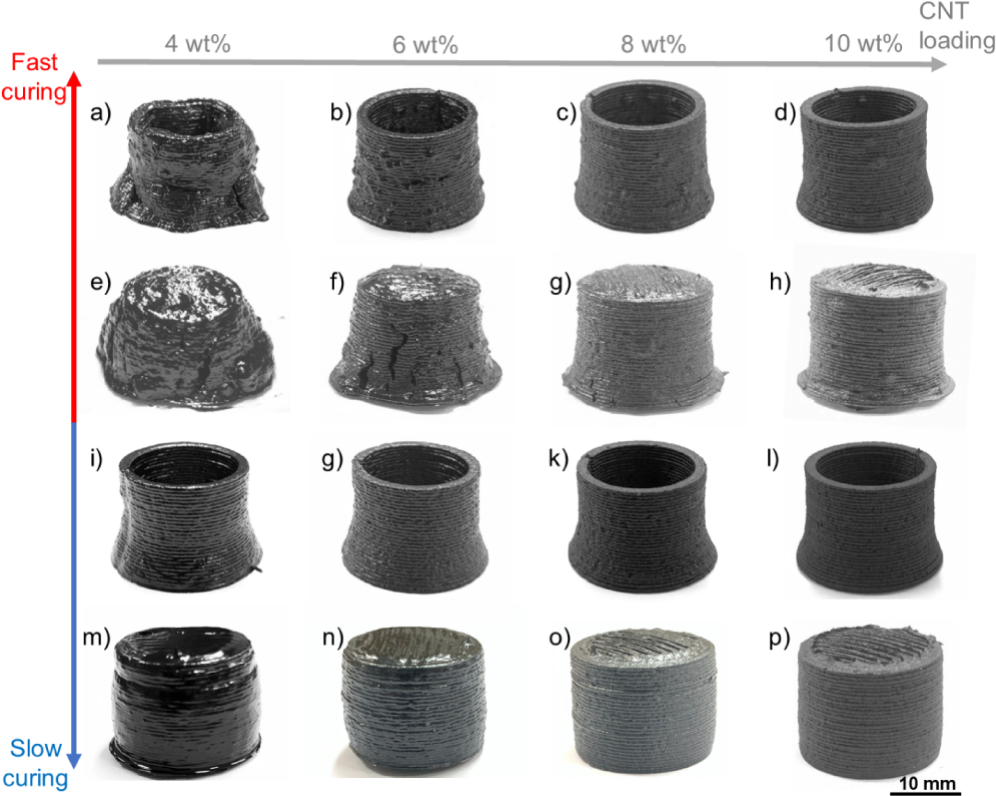
Effects of CNT loadings and curing cycles on the shape retention and quality of the printed CNT/phenolic thin walls (a–d and i–l) and dense towers (e–h and m–p): CNT/phenolic samples were cured via a fast curing schedule (a–h) and slow curing schedule (i–p). The data indicate that the bottom-right quadrant is most effective but also that the top-right quadrant is competitive.

Figure 3 presents CNT/phenolic thin walls and dense towers printed at different CNT loadings and cured via the fast and slow curing cycles. At the fast-curing cycle, the 4 wt % CNT/phenolic samples possessed severely distorted shapes (Figure 3a,e) due to the reduced viscosity at elevated temperature^22,23^ as well as the fast release of the byproducts.^34^ As shown in Figure 3a–h, shape retention of the cured CNT/phenolic samples gradually improved when CNT loading increased from 4 and 10 wt % because of the higher ink viscosities at elevated temperature.^22,23^

As shown in Figure 3i–p, all slowly cured samples possessed good shape integrity without visible cracks and bumps, except for the 4 wt % CNT/phenolic dense tower (Figure 3m). Due to the slow curing rate of the slow cycle, the viscosity at elevated temperature of the CNT/phenolic composites only experienced a slight reduction while the byproducts were released slowly, leading to the good shape integrity of most slowly cured samples. Regarding the 4 wt % CNT/phenolic dense towers, their reduced viscosity at elevated temperature was still insufficient to withstand their own weight, resulting in distortion and material accumulation at its bottom surface (Figure 3m).

Figure S2 shows the cryo-fracture surface of the slowly cured CNT/phenolic samples with a CNT loading from 4 to 10 wt %. There was no CNT agglomerates observed in any samples and the amount of CNTs at the fracture surface increased with increasing CNT loading. Importantly, excellent dispersion of CNTs in the phenolic matrix could be achieved even at high CNT loadings of 10 wt %. The result suggests that the dispersion process used in this work can successfully generate CNT/phenolic inks with good dispersion quality.

Because the printed 10 wt % CNT/phenolic samples had the best shape retention with good quality (Figure 3p), this ink formulation and curing process were used for further study in the next sections.

### 3.3 Effects of Sample Size on Shape Retention

**Figure 4:**
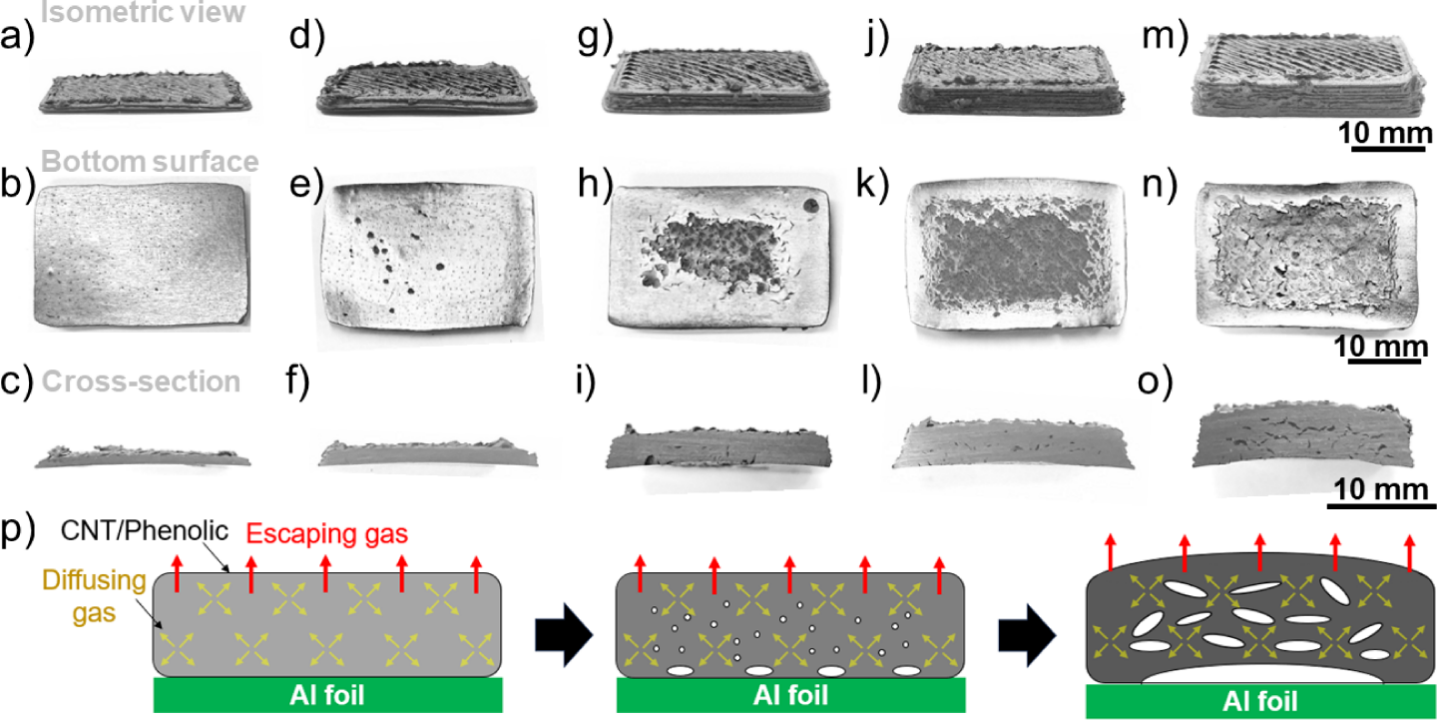
Isometric view, bottom surface, and cross-section of 10% CNT/phenolic samples printed on Al substrates and cured via the slow cycle. (a–c) 1 mm-thick samples, (d–f) 2 mm-thick samples, (g–i) 3 mm-thick samples, (j–l) 4 mm-thick samples, and (m–o) 5 mm-thick samples. (p) Schematic diagram of void formation in the 10 wt % CNT/phenolic structure printed Al substrates and cured via the slow cycle.

In this section, the effects of sample thickness and substrate types on the quality and mesostructure of the cured CNT/phenolic composite samples were investigated. Similar to the fast-cured neat phenolic resin, the fast-cured CNT/phenolic samples also possessed several large voids as well as volume expansion regardless of their thickness, as shown in Figure S3. Therefore, only the slow curing cycle was employed in this section. Figure 4 shows effects of thickness on the surface quality and cross-section of the 10 wt % CNT/phenolic samples printed on the Al substrates and cured via the slow cycle. As shown in Figure 4a–c, the 1 mm-thick samples possessed a smooth bottom surface and cross-section, suggesting that almost no byproducts were trapped inside the samples. When the sample thickness increased to 2 mm, there were some small holes occurring at the bottom surface (Figure 4d–e). However, no voids were observed at the sample cross-section (Figure 4f), indicating that most byproducts escape to the surrounding environment for this sample thickness.

For the samples with a thickness from 3 to 5 mm (Figures 4g–l,h–n, and i–o), a large concave region was formed at the center of the bottom surface of the printed samples, indicating the accumulation of the byproduct gas at this region. The increase in the size of the concave region with increasing sample thickness suggests that more gas was trapped at the bottom surface of the thicker samples. The smooth regions at the edges of the bottom sample surfaces indicate that there was a good attachment formed between the Al substrates and the printed samples in these regions. This generated a tight seal for byproduct gas accumulation and pressure buildup, leading to the bending of the 8 and 10 wt % CNT/phenolic samples, as shown in Figure 4l,o. Notably, there were no cracks at the surfaces of the samples, suggesting that the void formation and the sample bending occurred in the first stage of the curing process when the printed samples were still in the gel state. After the samples were completely transformed into the solid state at the end of the first reaction stage, the increased buildup pressure caused by the gas accumulation at the bottom sample surface led to the detachment of the samples from the Al substrate. This substrate detachment was observed in the heating oven during the sample heating from 90 to 100 °C. Additionally, more large voids were formed in the internal structure of the thicker printed parts, as shown in the cross-section of the composite samples in Figure 4i,l, and o.

The void formation mechanism of the 10 wt % CNT/phenolic samples printed on the Al substrates and cured via the slow cycle is schematically illustrated in Figure 4p. The reaction is volumetric and releases gases as a byproduct, but the migration of gas from the material is surface based. Thus, in samples with one thin dimension, the reaction byproducts are able to diffuse out; in thicker samples, the byproducts become trapped in the fully cured resin, resulting in voids, both in the internal structure and the bottom surface. (For the samples with low CNT loading, gas accumulation, and deformation can be reduced due to their lower viscosity, as shown in Figure S4, but this comes at the cost of shape retention.)

**Figure 5:**
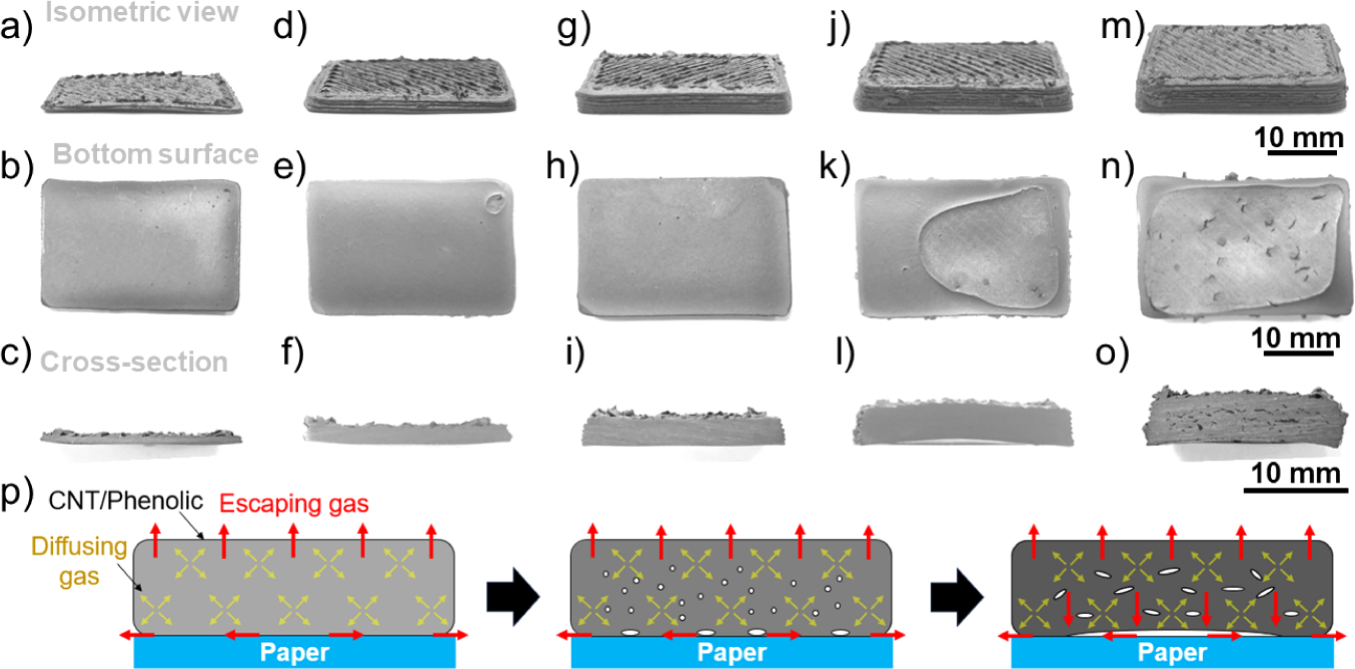
Isometric view, bottom surface, and cross-section of 10% CNT/phenolic samples printed on paper substrates and cured via the slow cycle. (a–c) 1 mm-thick samples, (d–f) 2 mm-thick samples, (g–i) 3 mm-thick samples, (j–l) 4 mm-thick samples, and (m–o) 5 mm-thick samples. (p) Schematic diagram of void formation in the 10 wt % CNT/phenolic structure printed Al substrates and cured via the slow cycle.

Figure 5 shows effects of thickness on surface quality and cross-section of the 10% CNT/phenolic samples printed on flexible paper substrates and cured via a slow cycle. As presented in Figure 5a–i, samples with a thickness from 1 to 3 mm had no voids. The results indicate that most byproduct gas can effectively escape from the printed structures in this thickness range; this is likely due to the flexible bottom surface, which allows gas to escape more easily than the rigid Al substrate. After the sample thickness increased to 4 mm, a large cavity occurred at the bottom surface of the printed samples (Figure 5k), indicating gas accumulation on the lower surface during the curing process, resulting in slight deformation but no internal voids, as shown in Figure 5l. Further increase in sample thickness to 5 mm led to the formation of many internal voids with a larger cavity at the bottom surface (Figure 5m–o).

The void formation mechanism of the 10 wt % CNT/phenolic samples printed on the flexible paper substrates and cured via the slow cycle is schematically illustrated in Figure 5p. Since the surfaces for most byproduct gas to escape to the surrounding environment were the top and bottom sample surfaces, the required travel distance of the gas to escape reduced by nearly twice, resulting in less gas trapped in the printed structure.

**Figure 6:**
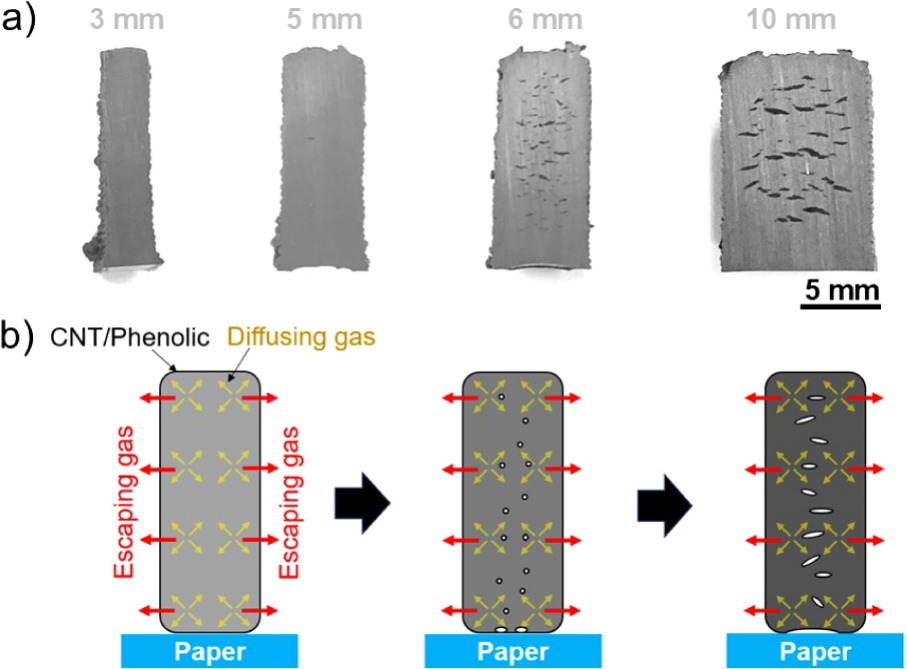
(a) Cross sections of 10% CNT/phenolic walls printed on paper substrates and cured via the slow cycle with different sample thicknesses. (b) Schematic diagram of void formation in the 10 wt % CNT/phenolic structure printed Al substrates and cured via the slow cycle.

The effects of sample thickness on the mesostructure of the 10 wt % CNT/phenolic samples is confirmed by printing and curing the standing wall samples using the slow cycle, as shown in Figure 6a. Interestingly, there were no visible voids at the cross-section of the phenolic-based samples with a thickness of up to 5 mm; in this case, the diffusion length scale to a free surface is 2.5 mm because there are no substrates restricting gas flow. Several small voids only occurred in the sample structures after the sample thickness increased to 6 mm, with larger voids present in the 10 mm sample (Figure S5). The results suggest that for 10 wt % CNT/phenolic composite parts, the diffusion length scale is on the order of 3 mm.

### 3.4 Electrical and Thermal Properties of the Printed CNT/Phenolic Composites

**Figure 7:**
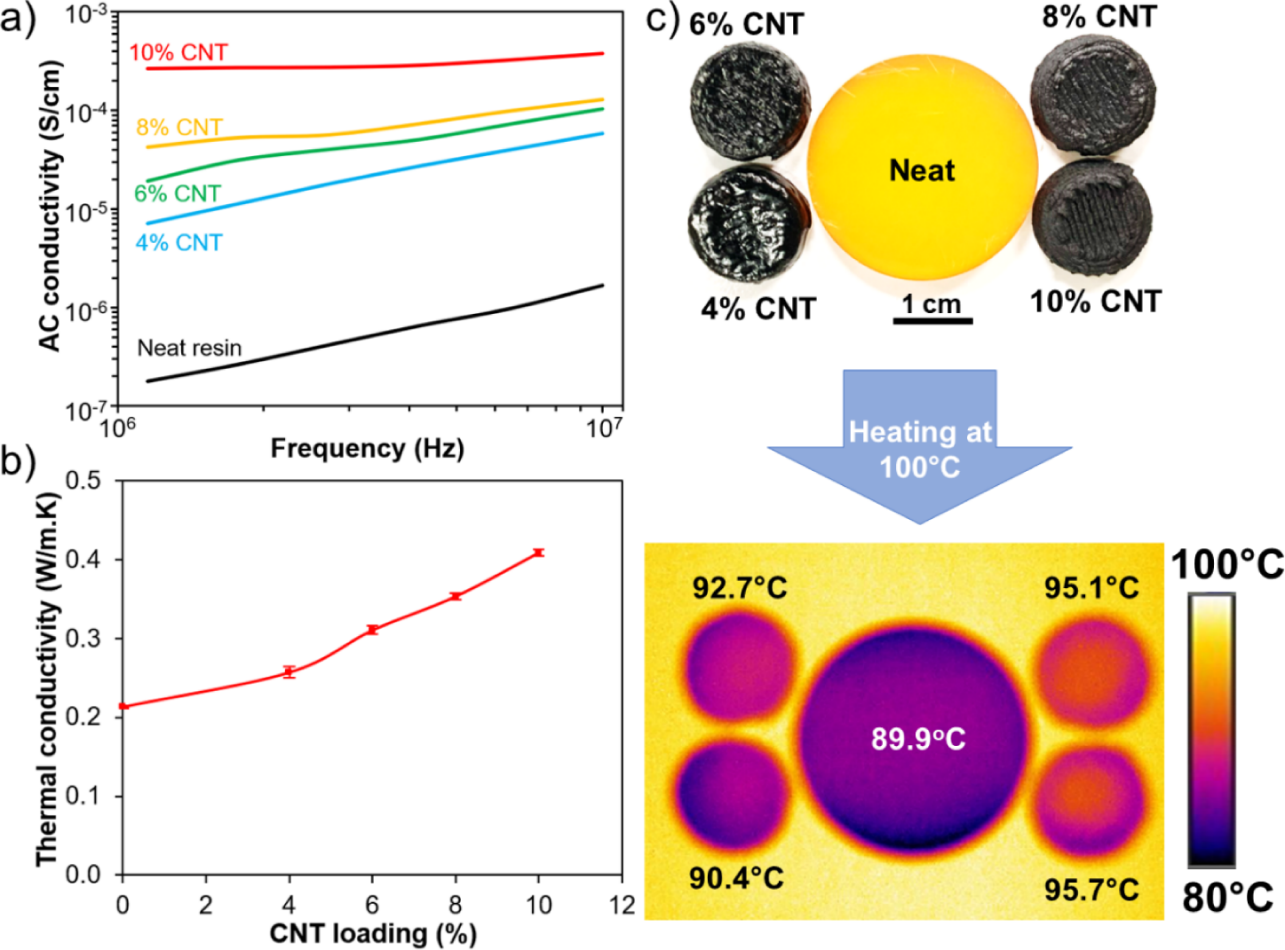
(a) AC conductivity, (b) thermal conductivity, and (c) infrared thermal images of the phenolic samples when the samples were employed as heat transfer medium. Neat phenolic samples were cast in a mold while the other samples were printed.

Figure 7a presents the AC electrical conductivity of the CNT/phenolic samples between the 1 and 10 MHz frequency range. The neat phenolic samples exhibited an electrical conductivity of less than 1.7 × 10^–6^ S/cm at all frequencies. With the incorporation of 4 wt % CNT, the electrical conductivity of the phenolic samples increased to 5.8 × 10^–5^ S/cm, while 10 wt % CNT/phenolic samples achieved an electrical conductivity of up to 3.8 × 10^–4^ S/cm at 10 MHz. These remarkable improvements are expected since CNTs with excellent electrical conductivity have been widely used as an effective electroconductive filler to enhance the electrical conductivity of insulating polymer matrices.^37,38^ Notably, electrical conductivity of all samples increased slightly with increasing frequency due to the increased mobility of electrons as expected.^11^

Figure 7b presents the thermal conductivities of the phenolic composite samples at varying compositions. The neat phenolic samples exhibited a low thermal conductivity of 0.213 W/m.K. Adding 4 wt % CNT raised the thermal conductivity by 20% to reach 0.257 W/m.K, since CNTs are also an effective thermoconductive filler.^37,38^ Increasing the CNT loading further improved the thermal conductivity, with the highest value of 0.408 W/m.K achieved at 10 wt % CNT loading. Figure 7c compares the surface temperature of different phenolic-based samples which were heated at 100 °C by a hot plate in 10 min. The neat phenolic samples possessed a surface temperature of 89.9 °C. By adding CNT fillers, the surface temperature of the phenolic composite samples increased gradually from 90.4 °C for 4 wt % CNT to 95.7 °C for 10 wt % CNT. The results are in good agreement with our previous reported work on printed CNT/silicone composites, indicating that CNTs can effectively enhance heat transfer and the thermal dissipation capabilities of the phenolic samples.^11^ Additionally, CNT/phenolic composites with better thermal conductivity might experience a more uniform curing process as heat transfer to their structures is more effective.

**Figure 8:**
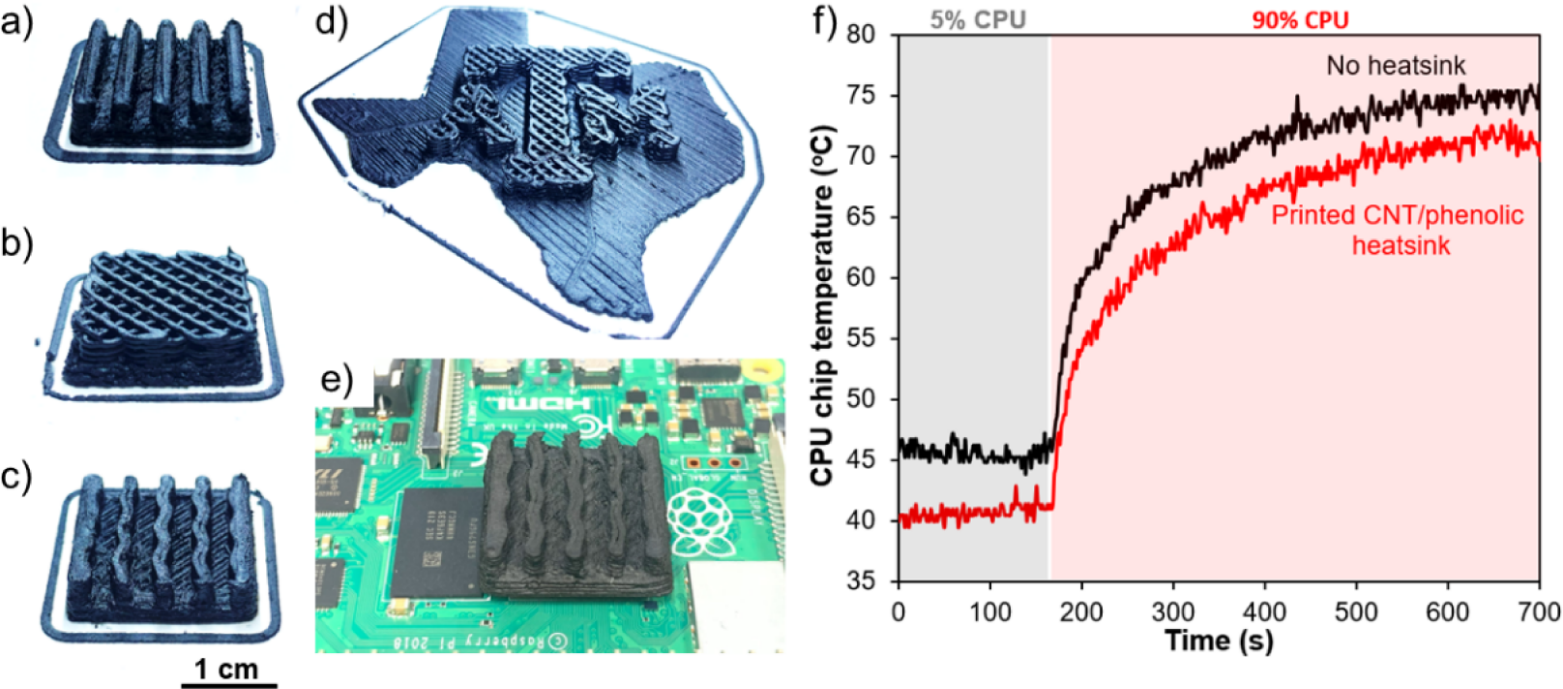
(a) 3D printing of 10 wt % CNT/phenolic heatsink with different designs and shapes: (a) Conventional plate fin heatsink, (b) nonconventional porous heatsink, (c) wavy plate fin heatsink, (d) porous heatsink with Texas A and M University logo shape, (e) wavy plate fin heatsink installed on the CPU chip of a Raspberry Pi board, and (f) temperature of the CPU chip at 5% and 90% CPU usage as a function of time.

Figure 8a–d presents 10 wt % CNT/phenolic heatsinks with various designs and configurations, such as a straight plate fin, a wavy plate fin, a porous design, and one with TAMU logo shape. The wall thickness of all heatsinks was less than 4 mm to minimize the defects caused by the byproducts during the curing process. To evaluate the thermal dissipation of the printed heatsinks, the wavy plate fin heatsink was installed on the CPU chip of the microcontroller board by using thermal tape (Figure 8e). Figure 8f compares the CPU chip temperature with and without heatsinks under 5% and 90% CPU usage scenarios. Interestingly, the printed CNT/phenolic heatsinks can effectively lower the chip temperature under the two working conditions. Without heatsink, the chip temperatures were approximately 45.7 and 74.9 °C when the CPU usage was 5% and 90%, respectively. However, the chip temperatures could drop to 40.4 °C when the CPU is at 5% usage and to 71.4 °C at 90% CPU usage by installing the CNT/phenolic heatsink. The results suggest that printed CNT/phenolic heatsinks can effectively address the overheating issue of modern electronic devices.

### 3.5 EMI Shielding Properties of the Printed CNT/Phenolic Composites

**Figure 9:**
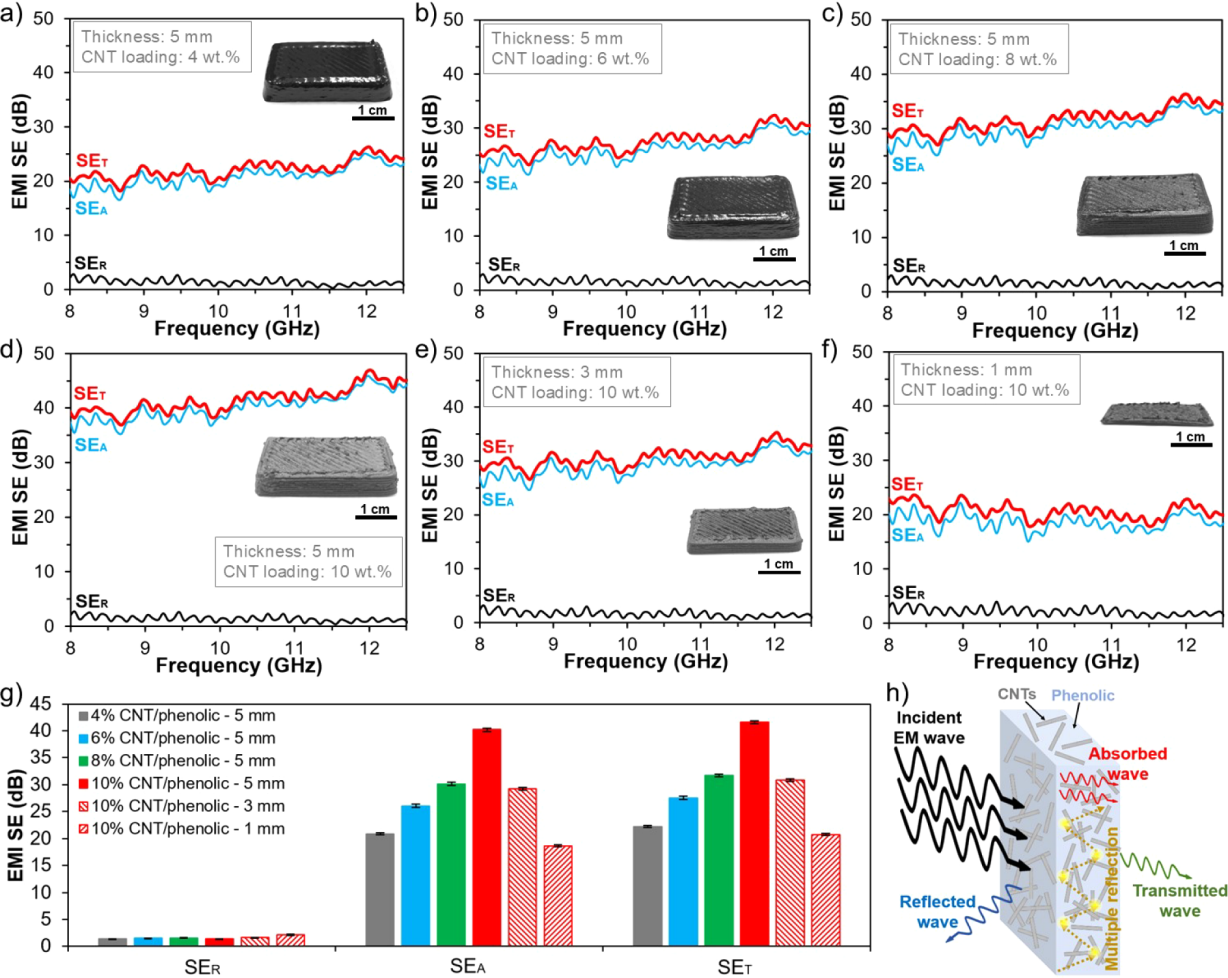
EMI shielding results of printed CNT/phenolic samples: (a) 4% CNT/phenolic (5 mm thickness), (b) 6% CNT/phenolic (5 mm thickness), (c) 8% CNT/phenolic (5 mm thickness), (d) 10% CNT/phenolic (5 mm thickness), (e) 10% CNT/phenolic (3 mm thickness), and (f) 6% CNT/phenolic (1 mm thickness). (g) Comparison of EMI shielding performance of printed CNT/phenolic samples with varying thickness and loading. (h) Schematic diagram of EMI shielding mechanism.

The reflection loss (SE_R_), absorption loss (SE_A_), and total EMI shielding effectiveness (SE_T_) of the printed CNT/phenolic samples were evaluated for various thicknesses and CNT loadings in this section. As shown in Figure 9, the EMI shielding performance of the CNT/phenolic composites exhibited a weak frequency dependence over the frequency range. The average SE_R_ values of all samples stayed within a narrow range between 1.3 and 2.2 dB, indicating the insignificant effects of sample thickness and CNT loading on this parameter. In contrast, both SE_A_ and SE_T_ were strongly dependent on the CNT loading and sample thickness. Specifically, the SE_A_ of the 5 mm-thick samples increased significantly from 20.87 dB for 4 wt % CNT to 40.22 dB for 10 wt % CNT, corresponding to an improvement of nearly 93%. Consequently, a SE_T_ of 41.6 dB and an EMI shielding efficiency of 99.99% were achieved for the 5 mm-thick samples at the CNT loading of 10 wt %. This excellent EMI shielding performance completely met the requirements for advanced shielding applications and was much better than other CNT composites fabricated by fused filament fabrication^39^ and DIW^40^ at the same CNT loading. With the reduction of the thickness to 1 mm, both SE_A_ and SE_T_ of the 10 wt % CNT/phenolic samples decreased significantly to 18.61 and 20.73 dB, respectively. The printed CNT/phenolic samples with larger thickness or higher CNT loading had better EMI shielding performance because their increased number of conductive CNT networks attenuated the EM waves more effectively.^7,41^

Notably, the total EMI shielding effectiveness of all samples was mainly contributed by the absorption loss, while the contribution of the reflection loss was negligible over all CNT loadings and sample thicknesses. At the CNT loading of 10 wt %, absorption loss made the largest contribution (96.7%) to the total EMI SE compared to the reflection loss (3.3%). The results suggest that shielding mechanism of the printed CNT/phenolic samples is the absorption-dominant.^6,10^ The EMI shielding mechanism of the printed CNT/phenolic samples is schematically illustrated in Figure 9h. When the incident EM waves reach the surface of the printed CNT/phenolic samples, it is partly reflected due to the impedance mismatch between the printed samples and air. When the rest of the EM wave propagates inside the printed composites, it is reflected, scattered, and absorbed by the numerous internal interfaces of the highly interconnected conductive CNT networks.^10,42^ Consequently, the EM waves are mainly absorbed and transformed into heat, resulting in the absorption dominant shielding mechanism.^10^ Finally, only negligible EM waves pass through the printed CNT/phenolic samples, therefore achieving an outstanding EMI shielding performance.

**Figure 10:**
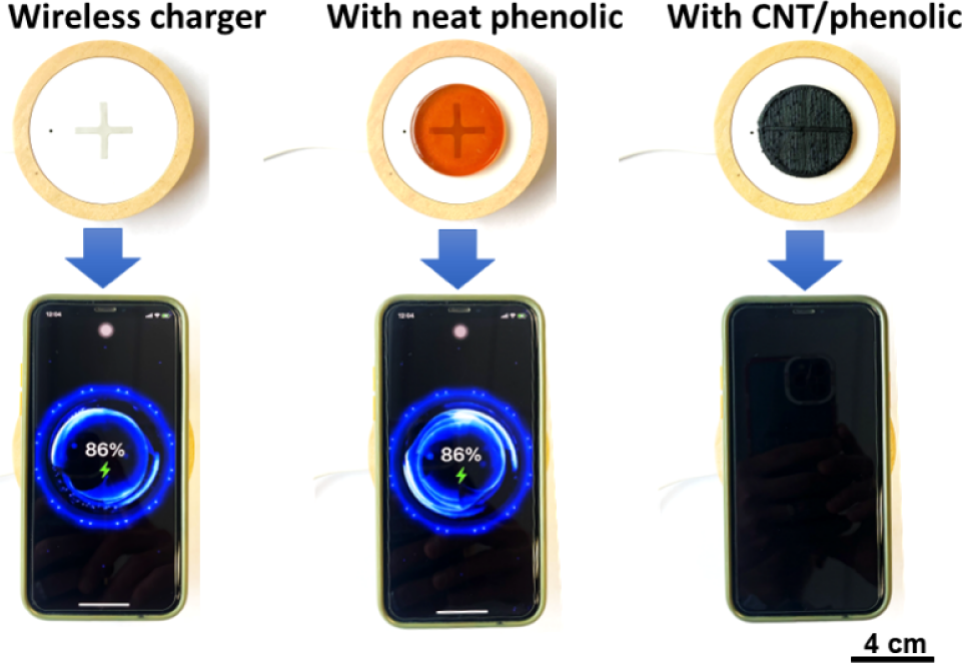
Demonstration of EMI shielding effectiveness of molded neat phenolic and printed 10% CNT/phenolic nanoparticles for wireless charging.

To evaluate the EMI shielding performance of the printed CNT/phenolic samples in practical conditions, a molded neat phenolic sample and a printed 10 wt % CNT/phenolic sample with the same thickness were inserted between a smartphone and a wireless charger, and the charging status of the smartphone was observed. As shown in Figure 10, the wireless charger covered with the neat phenolic sample could easily charge the phone, whereas there were no charging signals observed for the phone covered with the printed CNT/phenolic sample. The results suggest that the printed CNT/phenolic samples effectively blocked the wireless power transmission process, offering great potential to be used as EMI shielding materials in electronic packaging.

## 4 Conclusions

In conclusion, we produced CNT/phenolic composites via DIW 3D printing with thermal dissipation and an excellent EMI shielding performance. CNTs were employed as a viscosifier and a conductive nanofiller to improve printability and cure stability as well as thermal, electrical, and EMI shielding performance of the CNT/phenolic composites, respectively. It was found that printed 10 wt % CNT/phenolic thin-walled parts with highly dense structure can be fabricated by using a slow curing cycle and flexible substrate. Moreover, the electrical and thermal conductivities of the 10 wt % CNT/phenolic composites reach 3.8 × 10^–4^ S/cm at 10 MHz and 0.408 W/m·K, respectively, and an outstanding EMI shielding performance with a shielding efficiency of 99.99% can be achieved. This work demonstrates that high CNT loading, with one thin dimension, flexible substrates, and slow curing cycle, plays critical roles in fabricating high-performance 3D-printed CNT/phenolic composites for modern electronic applications.

## Supporting Information Available

Different stages of cross-linking reactions of PLENCO 14670; slow curing cycle of CNT/phenolic composites; TGA and DSC results for neat phenolic resin; cryo-fracture surface of printed CNT/phenolic with different CNT loading; isometric view and cross-section of 10% CNT/phenolic samples printed on Al substrates and cured by the fast cycle; bottom surface and cross-section of 5 mm-thick CNT/phenolic samples printed on Al substrates and cured by the slow cycle; isometric view, bottom surface, and cross-section of 5 mm-thick 10% CNT/phenolic samples printed on paper substrates and cured by the slow cycle (PDF)
