# Supplementary material for: 3D Printed Carbon Nanotube/Phenolic Composites for Thermal Dissipation and Electromagnetic Interference Shielding
Source: ACS Appl Mater Interfaces. 2024 Dec 4;16(50):69929–39. doi: 10.1021/acsami.4c17115 (PMC11660157; doi:10.1021/acsami.4c17115)
Supplement: Supplementary file 1 — am4c17115_si_001.pdf [file am4c17115_si_001.pdf]

Supporting Information

3D Printed Carbon Nanotube/ Phenolic Composites for Thermal Dissipation and Electromagnetic Interference Shielding

Thang Q. Tran, [†,§] *Sayyam Deshpande,* [†] *Smita Shivraj Dasari,* [†] *Kailash Arole,* [‡] *Denis Johnson,* [†] *Yufan Zhang,* [†] *Ethan M. Harkin,* [†] *Abdoulaye Djire,* [†, ‡] *Hang Li Seet,* [§] *Sharon Mui Ling Nai,* [§] *Micah J. Green* [*, †, ‡]

[†] Artie McFerrin Department of Chemical Engineering, Texas A&M University, College Station, TX, 77843, USA

[§] Singapore Institute of Manufacturing Technology (SIMTech), Agency for Science, Technology and Research (A*STAR), 5 Cleantech Loop, #01-01 Cleantech Two Block B, Singapore 636732, Republic of Singapore

[‡] Department of Materials Science and Engineering, Texas A&M University, College Station, TX, 77843, USA

*Corresponding author: E-mail: micah.green@tamu.edu (Micah J. Green)

Table S1. Different stages of cross-linking reactions of PLENCO 14670

Stage No.	Chemical reaction
1	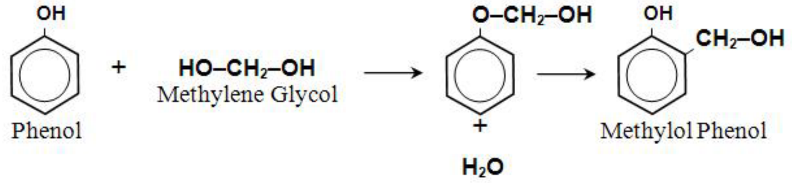
2	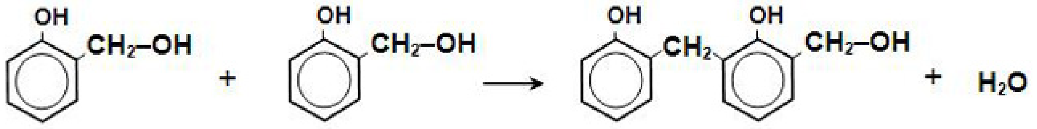
	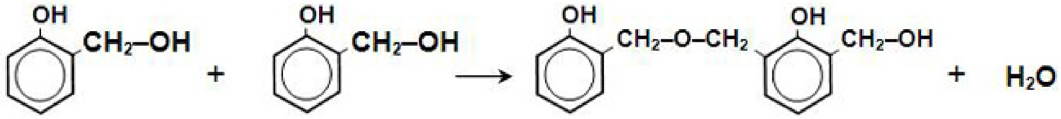
	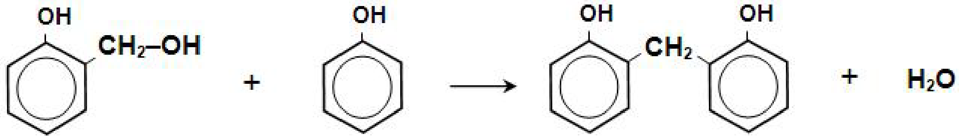

Table S2. Slow curing cycle of CNT/phenolic composites

**Temperature (°C)**	60	70	80	90	100	120	150
**Hold time (h)**	12	4	4	4	12	6	6

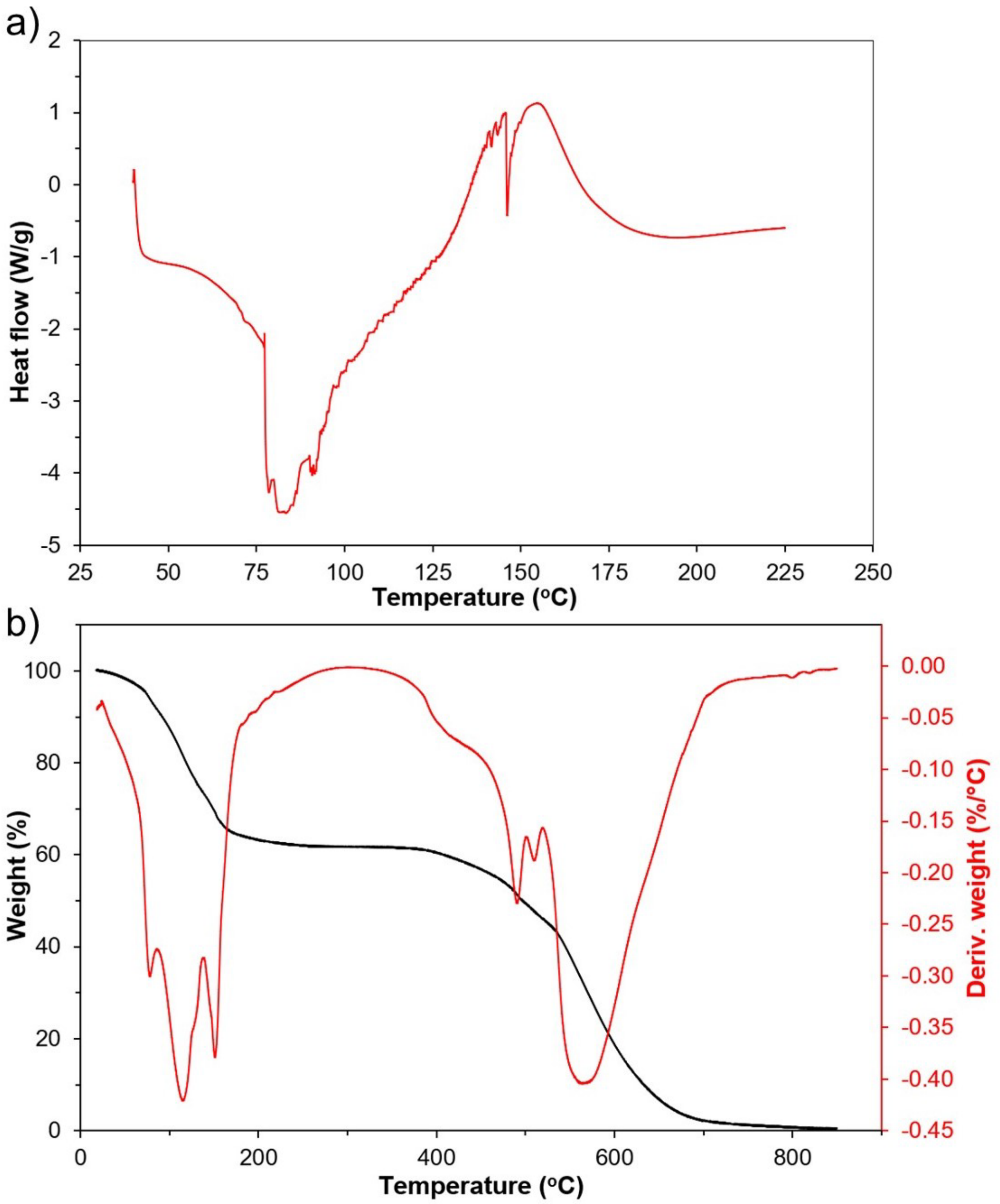

Figure S1. a) TGA and b) DSC results for neat phenolic resin

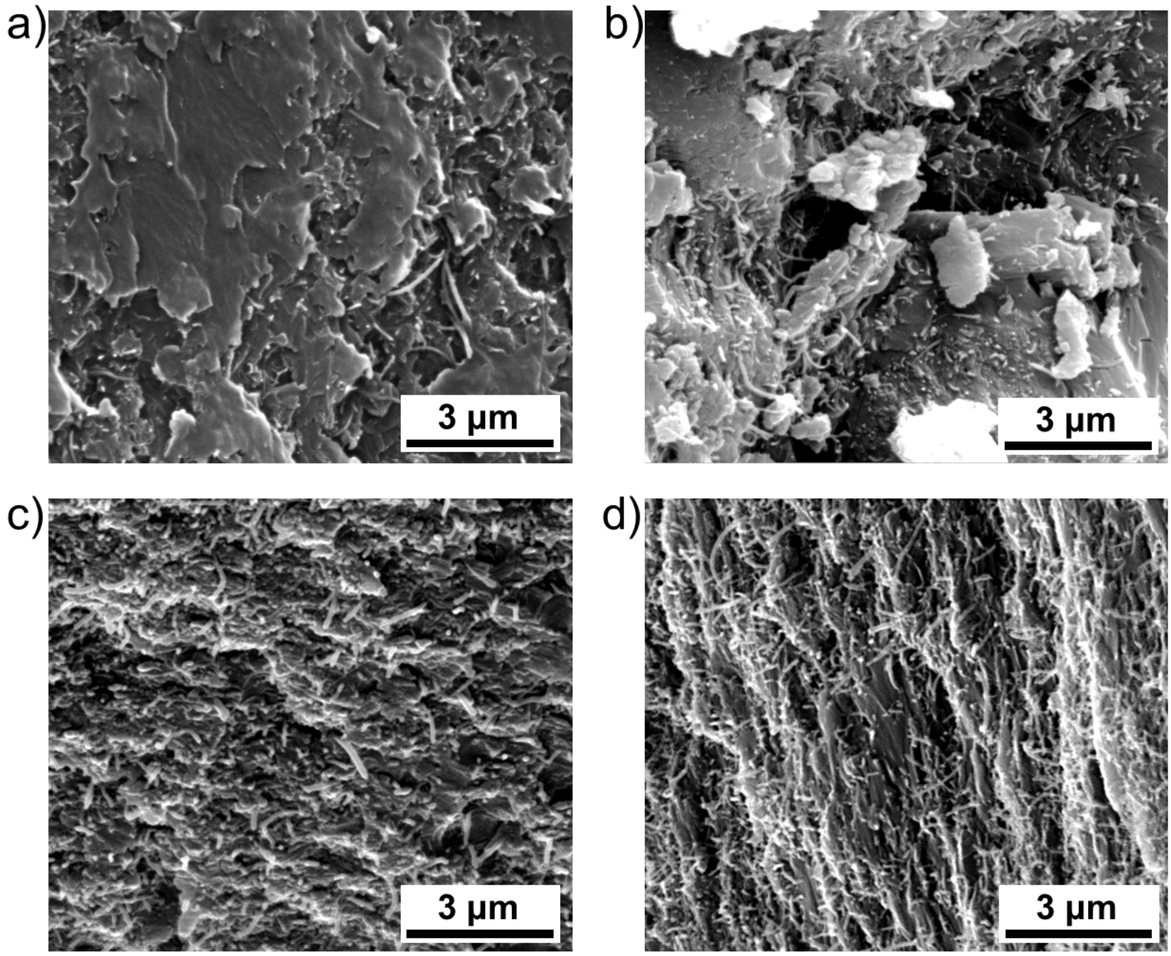

Figure S2. Cryo-fracture surface of printed CNT/phenolic with CNT loading of a) 4 wt.%; b) 6 wt.%, c) 8 wt.%, and d) 10 wt.%

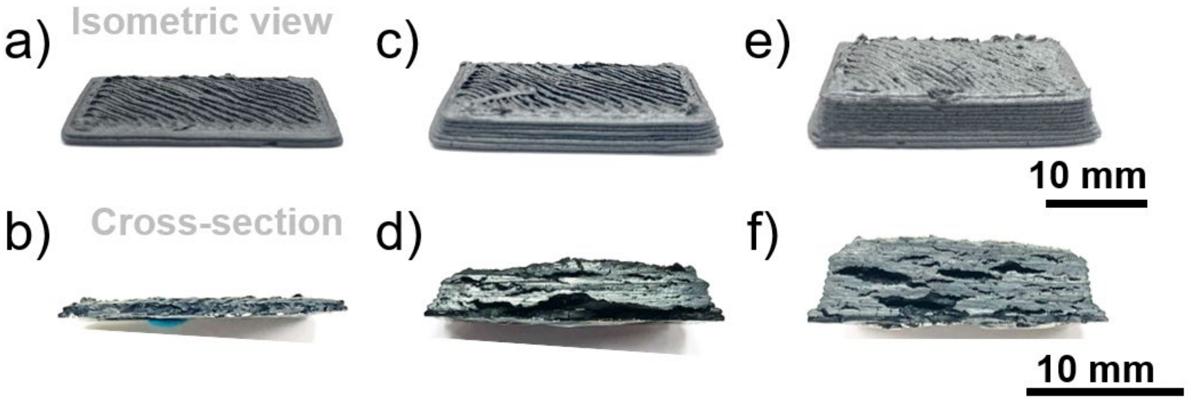

Figure S3. Isometric view and cross-section of 10% CNT/phenolic samples printed on Al substrates and cured by the fast cycle. (a, b) 1-mm-thick samples, (c, d) 3-mm-thick samples, and (e, f) 5-mm-thick samples.

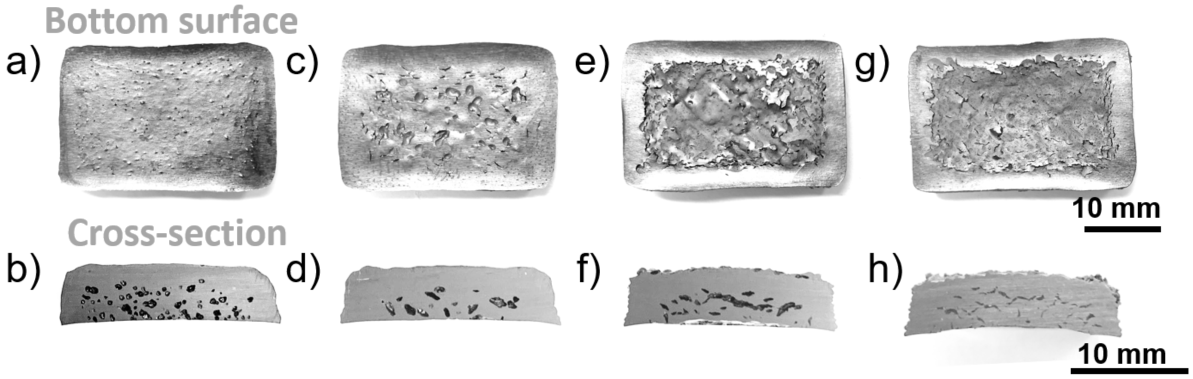

Figure S4. Bottom surface and cross-section of 5-mm-thick CNT/phenolic samples printed on Al substrates and cured by the slow cycle. (a, b) 4% CNT/phenolic, (c, d) 6% CNT/phenolic, (e, f) 8% CNT/phenolic, (g, h) 10% CNT/phenolic.

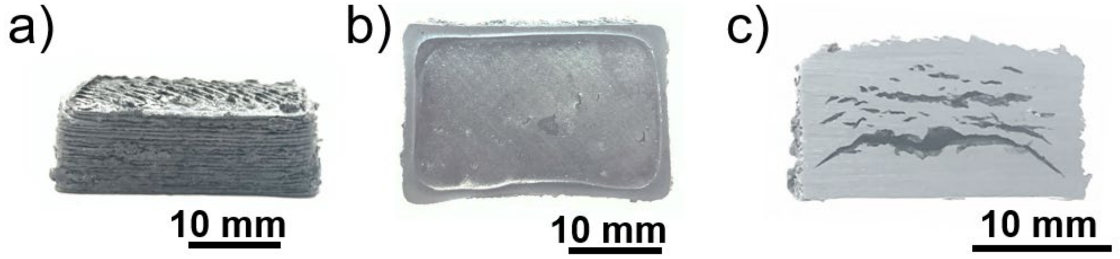

Figure S5. a) Isometric view, b) bottom surface, and c) cross-section of 5-mm-thick 10% CNT/phenolic samples printed on paper substrates and cured by the slow cycle.
